# Spatiotemporal Regulation and Functional Analysis of Circular RNAs in Skeletal Muscle and Subcutaneous Fat during Pig Growth

**DOI:** 10.3390/biology10090841

**Published:** 2021-08-30

**Authors:** Biao Li, Jinzeng Yang, Jun He, Yan Gong, Yu Xiao, Qinghua Zeng, Kang Xu, Yehui Duan, Jianhua He, Haiming Ma

**Affiliations:** 1 College of Animal Science and Technology, Hunan Agricultural University, Changsha 410128, China; 18874028597@163.com (B.L.); hejun@hunau.edu.cn (J.H.); 13910008175@139.com (Y.G.); xiaoyu1030189228@126.com (Y.X.); zhouwei2005@126.com (Q.Z.); jianhuahy@hunau.net (J.H.); 2 Department of Human Nutrition, Food and Animal Sciences, University of Hawaii at Manoa, Honolulu, HI 96822, USA; jinzeng@hawaii.edu; 3 Ningxiang Pig Farm of Dalong Livestock Technology Co. Ltd., Ningxiang 410600, China; 4 Laboratory of Animal Nutritional Physiology and Metabolic Process, Key Laboratory of Agroecological Processes in Subtropical Region, Institute of Subtropical Agriculture, Chinese Academy of Sciences, Changsha 410125, China; xukang2020@163.com (K.X.); duanyehui@isa.ac.cn (Y.D.)

**Keywords:** circular RNA, Ningxiang pig, Short Time-Series Expression Miner, skeletal muscle, subcutaneous fat

## Abstract

**Simple Summary:**

This study provides a clear and accurate dynamic profile of circRNAs in skeletal muscle and subcutaneous fat of Ningxiang pig. The results include high-quality genomic data, and help to elucidate the roles of circRNAs in regulation of protein and lipid synthesis and metabolism providing new ideas for further understanding of the molecular mechanisms governing pork quality traits.

**Abstract:**

Recently, thousands of circular RNAs have been reported in different pig breeds. However, researches on the temporal and spatial expression patterns of circRNA over the period of animal growth are limited. Here, we systematically analyzed circRNAs in skeletal muscle and subcutaneous fat in four growth time points (30 days, 90 days, 150 days and 210 days after birth) of a Chinese native pig breed, Ningxiang pigs. A total of 1171 differentially expressed (DE) circRNAs between muscle and fat were identified, including 562 upregulated and 609 downregulated circRNAs. KEGG pathway enrichment analysis of these DE circRNAs revealed that host genes were mainly involved in glycogen metabolism signaling pathways, muscle development signaling pathways such as ErbB pathway and adipocytokine signaling pathways and AMPK signaling pathways and fatty acid biosynthesis. The circRNAs have striking spatiotemporal specificity in the form of dynamic expression at 90 d. Short Time-Series Expression Miner analysis showed multiple model spectra that are significantly enriched with time changes in muscle and fat. Our findings provide new ideas and perspectives about the role of circular RNAs and their targeting relations with mRNA and miRNA in skeletal muscle and fat tissue during pig growth.

## 1. Introduction

After years of animal breeding programs, the lean meat percentage of commercial pigs has been considerably improved, but current living standards have also prompted consumers to seek pork with high meat quality. Chinese indigenous breeds are known for their meat quality, and there have been some reports of using Chinese native pigs as a biological model to study meat quality traits [1,2,3]. Ningxiang pig is well-known for its excellent meat quality and has become one of the four best-known local breeds in China. It has been reported that unsaturated fatty acids (UFAs) in Ningxiang pork account for up to 59.6% of all fatty acids [4]. Moreover, He et al. [5] found that the serum of Ningxiang pig contains more unsaturated lipids than that of lean pigs under the same environment and feeding conditions. Polyunsaturated fatty acids (PUFAs) are indispensable essential fatty acids for the body, while long-chain (≥C20) polyunsaturated fatty acids (LC-PUFAs) are highly biologically active forms of PUFAs, and their biological functions have become a significant research topic in international pig research communities [6,7]. This characteristic is a valuable trait of Ningxiang pig. It has been reported that the synthesis of unsaturated lipids is regulated by multiple genes, such as peroxisome proliferator activated receptor alpha (*PPARα*), fatty acid binding protein 4 (*FABP 4*), lipoprotein lipase (*LPL*) [8], CCAAT enhancer binding protein alpha (*C/EBPα*) [9], patatin-like phospholipase domain containing 3 (*PNPLA3*) [10], malic enzyme 1 (*ME1*) [11] and 2,4-dienoyl-CoA reductase 1 (*DECR1*) [12]. Therefore, as a research model, Ningxiang pig is of considerable significance for studies exploring genes related to meat quality traits and their expression patterns.

It is well known that biological processes are not only regulated by protein-coding RNA (mRNA) but also by noncoding RNA (ncRNA), such as microRNA (miRNA) and circular RNA (circRNA). CircRNAs are a group of new noncoding RNAs, characterized by the presence of a circular structure by 3′-end and the 5′-end covalent bonding. Functional circRNAs have been shown to have the functions of cytoplasmic microRNA sponge and nuclear transcription regulator in the regulation of gene expression [13]. There have been few reports that indicate large differences between fatty and lean pig breeds regarding the influence of circRNAs on pork quality. In a study of the subcutaneous adipose tissues of Large White Pigs and Laiwu Pigs, 275 differentially expressed circRNAs were identified. Among them, the target genes of circRNA_26852 and circRNA_11897 were indicated in regulation of adipocyte differentiation and lipid metabolism [14]. Adipose tissue is the primary energy storage organ and energy source of animals [15]. The amount of intramuscular fat (IMF) is related to the number of adipocytes and the ability to accumulate fat. Intramuscular fatty acids, especially polyunsaturated fatty acids, are important precursors of pork flavor substances that indirectly or directly affect indicators of meat quality, such as pH, water-holding capacity, tenderness, mouthfeel, and meat color [16]. However, the expression patterns and potential regulatory mechanisms of circRNAs related to meat quality or lipid anabolism in these organs across different developmental stages of pig have rarely been reported.

In this study, we first compared circRNA expression levels between two major metabolic tissues of Ningxiang pig, namely longissimus dorsi muscle and subcutaneous fat in four developmental stages (30, 90, 150, and 240 d after birth). The molecular characteristics, spatiotemporal expression patterns, potential functions and targeted miRNAs of the organ samples were systematically analyzed. To explore the dynamic expression pattern of circRNAs, we adopted a time-series analysis by STEM and constructed a network diagram of circRNA–miRNA–mRNA interactions that were significantly changed over time. Furthermore, screening circRNAs that affect fat metabolism throughout the developmental stages of pigs could significantly help to elucidate their biological and metabolic functions and relative importance.

## 2. Materials & Methods

### 2.1. Experimental Animals and Sample Collections

Twelve male purebred Ningxiang pigs were used in this study (all pigs were half-sibs). The experimental pigs were all provided by the Ningxiang pig breeding farm of Dalong Animal Husbandry Technology Co. Ltd. in Hunan Province, China. The 12 pigs were examined at each of the four developmental stages in this study, including 30 d (30 d of age), 90 d (90 d of age), 150 d (150 d of age) and 210 d (210 d of age). Three biological repeats were employed per time point. Under standard animal care and management conditions, pigs were fed three times per day on a regular basis, with free access to water. Next, 3 healthy individuals with no disease and similar body weights were randomly selected in the 4 age groups from 30 d to 210 d in accordance with standard procedures. The pigs sacrificed by exsanguination under isofluraneanesthesia at the surgical table (4.5% tidal volume by mask). The longissimus dorsi muscle and subcutaneous fat samples from the fourth to last rib were collected within 30 min after slaughter, frozen in liquid nitrogen, and stored at −80 °C.

### 2.2. RNA Extraction

Total RNA was extracted from the tissue using RNA Purification Reagent according the manufacturer’s instructions (Invitrogen, California, USA), and genomic DNA was removed using rDNaseIRNase-free (TaKaRa Bio Inc, Dalian, China). RNA quality was verified using a 2100 Bioanalyzer (Agilent Technologies, Santa Clara, CA, USA) and an ND-2000 spectrophotometer (Thermo Fisher Scientific, Waltham, MA, USA). Only high-quality RNA samples (OD260/280 = 1.8~2.2, OD260/230 ≥ 2.0, RIN ≥ 6.5, 28S:18S ≥ 1.0, >10 μg) were employed to construct a sequencing library.

### 2.3. Library Preparation and Illumina HiSeq 4000 Sequencing

The RNA-seq transcriptome strand library was prepared by following the TruSeq^TM^ Stranded Total RNA kit from Illumina (San Diego, CA, USA) using 5 μg of total RNA. Briefly, ribosomal RNA (rRNA) depletion instead of poly(A) purification was performed using an IlluminaRibo-Zero Magnetic kit, first employing fragmentation by fragmentation buffer. The first-stranded cDNA was synthesized with random hexamerprimers. Then, the RNA template was removed and a replacement strand was synthesized, and dUTP was used instead of dTTP to generate ds cDNA. AMPure XP beads are used to isolate ds cDNA from the second-strand reaction mixture. A single “A” nucleotide is added to the 3′ end of these blunt-ended fragments to prevent them from connecting to each other during the linker ligation reaction. Finally, connect multiple index adapters to the ends of the ds cDNA. The cDNA target fragments of 200–300 bp were selected for size selection on 2% UltraAgarose, and then 15 PCR cycles of PCR amplification were performed using Phusion DNA polymerase (NEB). After quantification by TBS380, the paired-end RNA sequencing library was sequenced with Illumina HiSeq 4000. In addition, 3 μg of total RNA was connected to the sequencing adapter using TruSeqTM Small RNA Sample Prep Kit (Illumina, San Diego, CA, USA). Subsequently, cDNA was synthesized by reverse transcription and amplified with 12 PCR cycles to generate a library. After that, Shanghai Meiji Biomedical Biotechnology Co., Ltd. will conduct deep sequencing (Shanghai, China).

### 2.4. Read Mapping and Transcriptome Assembly

The raw paired-end reads are trimmed and quality controlled by SeqPrep (https://github.com/jstjohn/SeqPrep, accessed on 5 October 2020) using default parameters. Next, the HISAT2 (https://ccb.jhu.edu/software/hisat2/index.shtml, accessed on 10 October 2020) software was used to align the clean reads with the reference genome (accession number PPJNA531381) in directed mode. The mapped reads of each sample were assembled by StringTie (https://ccb.jhu.edu/software/stringtie/index.shtml, accessed on 10 October 2020) using a reference-based method.

### 2.5. Identification of circRNAs

CIRI2 (CircRNA Identifier 2) is used in conjunction with find_circ to identify circRNA. Scan the SAM file twice and collect enough information to identify and characterize circRNA. CIRI 2 detects junction reads with PCC signals that reflect candidate circRNA. Use paired-end mapping (PEM) and GT-AG splicing signals to achieve preliminary filtering. After the clustering junction reads and recording each circRNA candidate, CIRI 2 scans the SAM alignment again to detect the junction reads and performs further filtering to eliminate false positive candidates that may be caused by mismapped reads of homologous genes or repetitive sequences. Then the identified circRNAs are output together with the annotation information. The expression level of each circRNA according to the Reads per million mapped reads (RPM) method was calculated.

### 2.6. Differential Expression Analysis and Functional Enrichment

Significantly differentially expressed (DE) circRNAs were extracted with |log2FC| > 1 and *p*-adjust ≤ 0.05 by Deseq2. In addition, functional enrichment analysis, KEGG analysis was performed to identify which DECs were significantly enriched in metabolic pathways at *p*-values ≤ 0.05 compared with the whole-transcriptome background. KEGG pathway analysis was performed using KOBAS.

### 2.7. Time-Series Analysis

Time-series analysis was performed by STEM (Short Time-Series Expression Miner) [17]. Time-series analysis studies the dynamic behavior of gene expression and measures a series of processes that have strong correlations with time points. This analysis produces a color for backgrounds with significant profiles and produces a white background for profiles that are not significant. Model profiles of the same color belong to the same cluster of profiles. Significantly enriched model profiles are indicated by different colors (Benjamini–Hochberg-adjusted *p*-values ≤ 0.05). The corrected *p*-values are sorted from small to large.

### 2.8. Analysis of circRNAs Regulatory Network

To reveal the role and interactions among ncRNAs and mRNAs, we constructed a ncRNA–mRNA regulatory network. MiRanda was used to predict the circRNA–miRNA–mRNA pairs (score cutoff ≥ 160 and energy cutoff ≤ −20). The co-expression network of circRNA–miRNA–mRNA was constructed using Cytoscape software (v3.2.1) to investigate the function of key circRNAs [18].

### 2.9. Validation of Expression by PCR

In order to verify the identified circRNAs in Ningxiang pigs, total RNA was extracted using Animal Total RNA Kit (Tiangen, Beijing, China) and treated with ribonuclease R (RNase R). RevertAid First Strand cDNA Synthesis Kit (Thermo Scientific, Waltham, MA, USA) was used to synthesize cDNA by reverse transcription. We used the “outward” strategy to design different PCR primers to exclude linear mRNA from amplification (synthesized by Shenggong Biotechnology Co., Ltd., Shanghai, China). The PCR products were verified by Sanger sequencing.

### 2.10. Fluorescence In Situ Hybridization (FISH)

Muscle samples fixed with 4% paraformaldehyde were cut in a sagittal direction into 10-um sections and dewaxed in paraffin sections in water. The sections were boiled in repair solution for 10 min. Proteinase K (20 μg/mL) was added dropwise and allowed to react with the samples at 37 °C for 30 min, then washed away by rinsing with PBS for 5 min 3 times. Then, the prehybridization solution was added and incubated at 37 °C for 1 h. We added a certain concentration of Chr01_143206410_143210729 probe (5′-GCTTCCTGTTTTTTACTTGGGCTGTTAG-3′) (General Biol, China) to the hybridization solution to hybridize overnight at 37 °C. The nuclei stained with DAPI (Servicebio, Wuhan, China) appeared blue under ultraviolet excitation, and positive expression was indicated by red fluorescence. The images were observed using an upright fluorescence microscope (Nikon, Tokyo, Japan).

### 2.11. Statistical Analysis

Based on the differential gene expression analysis of negative binomial distribution, DESeq2 is used, which is a method of differential analysis of count data. Use shrinkage estimation to estimate the dispersion and fold changes to improve the stability and interpretability of the estimation. This allows a more quantitative analysis to focus on intensity, not just the existence of differential expression. Expression level difference analysis is a statistical inference process to determine the occurrence of differential expression of all detected genes. Due to the large number of genes involved, statistical tests need to be performed multiple times (the number of genes to be tested). In order to control the false discover rate, the *p* value obtained from statistical tests needs to be corrected, so we performed multiple correct tests. Then the p-adjust value smaller than 0.05 was significant. In DESeq2, Wald test is usually used for hypothesis testing when comparing two groups. We used the R script to perform KEGG PATHWAY enrichment analysis on the genes in the gene set. When the *p* value < 0.05, the KEGG PATHWAY function is considered to be significantly enriched.

## 3. Results

### 3.1. Identifications and Characteristics of circRNAs

**Figure 1:**
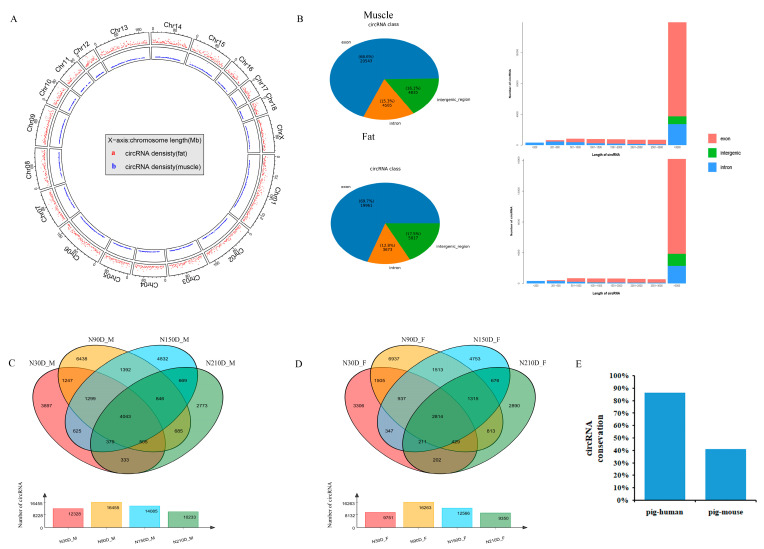
Identification and feature of circRNAs in Ningxiang pig. (**A**) Distribution of circRNAs in each chromosome. (**B**) Classification and Length distribution of circRNAs in Ningxiang pig skeletal muscle and subcutaneous fat. (**C**) Venn diagram of circRNAs identified at four development points in skeletal muscle of Ningxiang pigs. (**D**) Venn diagram of circRNAs identified at four development points in subcutaneous fat of Ningxiang pigs. (**E**) Sequence conservation of Ningxiang pig circRNA compared with human and mouse ones, respectively.

To perform comprehensive profiling of circular RNA in pig, we performed total RNA sequencing in two major tissues of lipid anabolic metabolism (fat and skeletal muscle) and across four developmental stages (30 d, 90 d, 150 d and 210 d after birth). In this study, after low-quality and adapter-polluted reads were first removed from the raw data, the Q30 value of the clean reads of the two tissues at 30 d, 90 d, 150 d, and 210 d was observed to be greater than 95% (Table 1). Among the samples, as mapped with the Ningxiang pig genome (accession number PPJNA531381) assembled by our research group, the mapping rate of each sample exceeded 92%. In addition, CIRI2 was used for sequence segmentation alignment to accurately identify circRNAs. We identified 45,892 unique circRNAs in all assessed biological tissues (Supplemental Table S1). Analysis of chromosome distribution indicated that the identified circRNAs were transcribed widely and unevenly on the chromosome. Compared with other chromosomes, most of the circRNAs to be identified were distributed on chromosome 1, chromosome 6 and chromosome 13, which is in keeping with the characteristics of circRNAs reported by others [19]. In addition, we also detected circRNAs in the sex chromosomes (Figure 1A). As shown in previous studies, most circRNAs contain multiple exons, and some circRNAs also retain introns. In this study, most of the circRNAs identified (approximately 68.6% to 69.7%) were exons followed by intergenic circRNAs (approximately 16.1% to 17.5%), and only a small portion (approximately 12.8% to 15.3%) were located in introns. The length analysis showed that most circRNAs were longer than 3000 bp (Figure 1B). We further constructed Venn diagrams of the identified circRNAs at four time points (Figure 1C,D). It was found that a total of 9202 circRNAs were co-expressed in the four time periods. However, the number of circRNAs identified at 90 d was the largest, and the number of circRNAs that were uniquely expressed at 90 d was notably greater than that at other time points. CircRNAs also showed tissue-specific and time-specific expression in skeletal muscle and fat during the development of Ningxiang pig.

Several studies have shown that some circRNAs are sequence conservative among humans and mice [20,21,22]. Indeed, 25.45% and 87.44% the identified S. scrofa circRNAs were mapped to mice and humans, respectively. These findings suggest that the production of circRNAs from orthologous genes of pig, human and mouse are moderately conserved and might have conserved functions. Blast method was used to compare the identified circRNAs of Ningxiang pig to human and mouse circRNAs. We found that 86% and 41% Ningxiang pig circRNAs have orthologs in humans and mice, respectively (Figure 1E and Supplemental Table S2). The homology between Ningxiang pig circRNA and human is higher than that of mouse, which is consistent with previous reports.

### 3.2. Expression Patterns of circRNAs during Muscle Growth

**Figure 2:**
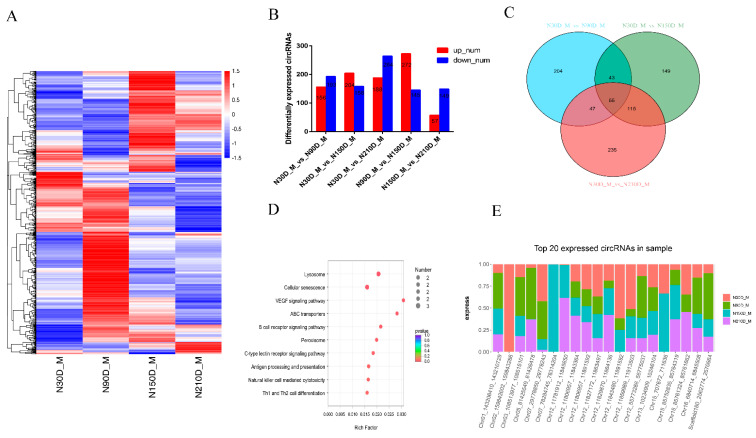
Differentially expressed (DE) circRNAs and their expression modes in skeletal muscle. (**A**) Heat map of all DE circRNAs among the five compared groups (30 d vs. 90 d, 30 d vs. 150 d, 30 d vs. 210 d, 90 d vs. 150 d, and 150 d vs. 210 d groups). (**B**) Number of differentially expressed circRNAs in skeletal muscle vs. means versus. (**C**) The number of common DE circRNAs in skeletal muscle. (**D**) KEGG pathway analysis of common DE genes in skeletal muscle. The top 10 enriched KEGG pathways ranked by *p*-values are shown. (**E**) Top circRNAs expressed in skeletal muscle.

Intramuscular fat (IMF) content and fatty acid composition are important meat quality characteristics [23]. However, the molecular mechanisms regulating intramuscular fat accumulation and fatty acid composition have not been fully elucidated. To understand the regulatory roles of circRNAs in skeletal muscle, we analyzed the expression profile of circRNAs in skeletal muscle at 30, 90, 150 and 210 d after birth. This analysis can assess the dynamic changes in circRNA expression from lactation to fattening and identify circRNAs that may be related to IMF deposition and fatty acid composition. First, we designed five comparison groups (1:30 d vs. 90 d; 2: 30d vs. 150 d; 3: 30d vs. 210 d; 4: 90 d vs. 150 d; and 5: 150 d vs. 210 d) to characterize the differential expression of circRNAs (DECs) during development. Differentially expressed circRNAs were identified by DEseq2 with *p*-adjust ≤ 0.05 and fold-changes > 2 between any two groups. A total of 1786 circRNAs were defined as DECs among these comparison groups (Figure 2A).

We attempted to evaluate the potential functions of differentially expressed circRNAs (DECs) between two closed time points. Compared with piglets (30 d after birth), among these DECs, 156 circRNAs were upregulated at 90 d, 204 circRNAs were upregulated at 150 d, and 188 circRNAs were upregulated at 210 d (Figure 2B and Supplemental Table S3). Under the assumption that the functions of circRNAs are related to the functions of their host genes, we performed KEGG pathway analysis to predict the functions of DECs during the growth and development of skeletal muscle. KEGG pathway analysis showed that, compared to the lactation period (30 d), the biological functions of these three closed time points were different. For example, in skeletal muscle, significantly upregulated pathways at 90 d are primarily involved in such processes as propanoate metabolism, valine, leucine and isoleucine degradation, and starch and sucrose metabolism; the significantly upregulated pathways at 150 d are primarily involved in such processes as ubiquitin-mediated proteolysis, cellular senescence, and starch and sucrose metabolism; the significantly upregulated pathways at 210 d are primarily related to genetic information processing, including such processes as RNA degradation, ubiquitin-mediated proteolysis, and protein processing in the endoplasmic reticulum. However, the downregulated pathways of these three groups of closed time points are all related to lipid metabolism and transport, including such processes as fatty acid biosynthesis, primary bile acid biosynthesis, and unsaturated fatty acid and peroxisome biosynthesis (*p* < 0.05, Figure 1 and Supplemental Table S4). Overall, studies of the host genes of DECs at various time points indicate that the circularization of these genes may be important for skeletal muscle function. Among these circRNAs, we also found that 55 were identified as common DECs throughout the muscle development process (Figure 2C and Supplemental Table S5). These common DECs play important roles in muscle development. KEGG pathway analysis indicated that these common DECs were mainly enriched in lysosomes, cellular senescence, the VEGF signaling pathway, and ABC transporters (Figure 2D).

To study the role of circRNAs in the transformation of skeletal muscle function, in addition to 30 d vs. 90 d, we added 90 d vs. 150 d and 150 d vs. 210 d comparisons. The results present circRNAs that were up- or downregulated between two consecutive time points during muscle growth. Between 90 d and 150 d, the significantly upregulated pathways were primarily involved in such processes as cell longevity, EGFR tyrosine kinase inhibitor resistance, and lysine degradation, and the downregulated pathways were primarily involved in such processes as valine, leucine and isoleucine degradation, ubiquitin-mediated proteolysis, and regulation of actin cytoskeleton. Compared with the results obtained at 150 d, the pathways upregulated at 210 d were determined to be primarily related to rheumatoid arthritis, antigen processing and presentation, and cytokine-cytokine receptor interactions, and downregulated pathways were observed to be primarily related to such metabolic pathways as starch and sucrose metabolism, ketone body synthesis and degradation, and valine, leucine and isoleucine degradation (*p* < 0.05, Figure S1 and Supplemental Table S4). These up- and downregulated circRNAs at different time points may be associated with regulating the initiation or termination of growth and/or physiological processes at specific developmental stages. We also focused on the changing rules of the 20 most abundant circRNAs in skeletal muscle. Notably, several of the most abundant circRNAs in skeletal muscle originate from protein-coding genes with pivotal roles in skeletal muscle growth, intramuscular fat deposition, and intramuscular sugar metabolism (e.g., *MYH2*, *MYH6*, *TTN*, *PFKFB1*, *TNNI2*, *MYBPC1*, and *PRKAR1A*) (Figure 2E and Supplemental Table S6).

### 3.3. Expression Patterns of circRNAs in Adipose Tissue

**Figure 3:**
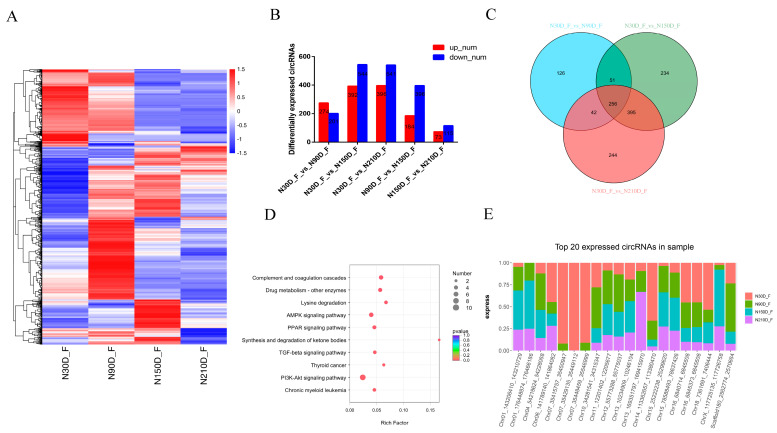
Differentially expressed (DE) circRNAs and their expression modes in subcutaneous fat. (**A**) Heat map of all DE circRNAs among the five compared groups (30 d vs. 90 d, 30 d vs. 150 d, 30 d vs. 210 d, 90 d vs. 150 d, and 150 d vs. 210 d groups). (**B**) Number of differentially expressed circRNAs in subcutaneous fat. vs. means versus. (**C**) The number of common DE circRNAs in subcutaneous fat. (**D**) KEGG pathway analysis of common DE genes in subcutaneous fat. The top 10 enriched KEGG pathways ranked by *p*-values are shown. (**E**) Top circRNAs expressed in subcutaneous fat.

Adipose tissue is primarily composed of fatty acids (FAs) and triglycerides, which play important roles in the quality of meat [24]. However, the type and proportion of FAs are also affected by such factors as breed and genetic conditions [25,26]. We clustered 3116 differential circRNAs identified in the five comparison groups to obtain an overview of DECs in adipose tissue (Figure 3A). Among these closed time points, we detected 274 circRNAs upregulated at 90 d, 392 circRNAs upregulated at 150 d, and 396 circRNAs upregulated at 210 d compared to 30 d (Figure 3B and Supplemental Table S7). KEGG analysis showed that compared with the lactation period (30 d), the upregulated pathways at these three closed time points were significantly enriched in the AMPK signaling pathway. These host genes primarily include *PIK3R1*, *IGF1R*, *PPP2R3A*, *PPARG*, *RPS6KB1*, *ACACA*, and *PPP2R3A*, which encode proteins associated with adipocyte development and lipid anabolism. However, the downregulated KEGG pathways of these three groups of closed time points are all related to such pathways as complement and coagulation cascades, fatty acid biosynthesis, and steroid hormone biosynthesis. In addition, between 90 d and 150 d, significantly upregulated pathways are primarily involved in such pathways as synthesis and degradation of ketone bodies, synthesis of basal transcription factors, and acute myeloid leukemia, and downregulated pathways are primarily involved in such pathways as complement and coagulation cascades, steroid hormone biosynthesis, and primary bile acid biosynthesis. Compared to 150 d, the upregulated pathways at 210 d are primarily related to such metabolic pathways as the citrate cycle (TCA cycle), lysine degradation and glutathione metabolism, and downregulated pathways are primarily related to such pathways as protein processing in the endoplasmic reticulum, starch and sucrose metabolism, and adhesion junctions (*p* < 0.05, Figure S1 and Supplemental Table S4).

Compared to piglets at 30 d, 256 circRNAs were identified as common DE genes during the entire development process of adipose tissue, indicating that the KEGG pathways were primarily enriched in the complement and coagulation cascades, lysine degradation, the PPAR signaling pathway, the TGF-beta signaling pathway, the AMPK signaling pathway, and the PI3K-Akt signaling pathway (Figure 3C,D and Supplemental Table S8). Among the most abundant circRNAs expressed in adipose tissue, several circRNAs derived from protein-coding genes that affect the key roles of adipose tissue development and fatty acid metabolism are highly expressed in adipose tissue (e.g., NADP-dependent malic enzyme (*ME1*), protein kinase A type 1-α regulatory subunit (*PRKAR1A*), and 6-phosphofructo-2-kinase/fructose-2,6- bisphosphatase 1 (*PFKFB1*) (Figure 3E and Supplemental Table S9).

### 3.4. Differentially Expressed circRNAs between Muscle and Adipose Tissue

**Figure 4:**
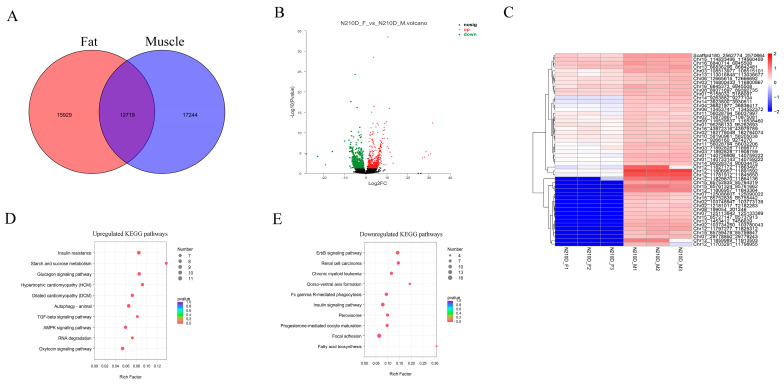
Expression analysis of circRNAs between skeletal muscle and subcutaneous fat of Ningxiang pig. (**A**) Venn chart of circRNAs detected in skeletal muscle and subcutaneous fat of Ningxiang pig. (**B**) Volcano plot of all DE circRNAs between skeletal muscle and subcutaneous fat. The x-axis represents the value of log2 (muscle/fat) and the y-axis represents the value of -log10 (*p*-value). Green, black, and red dots represent downregulated, unchanged, and upregulated DEcircRNAs, respectively, between the skeletal muscle and subcutaneous fat groups. (**C**) Heatmap illustrating the relative expression of top 50 DE circRNAs from three 210 d skeletal muscle and three 210 d subcutaneous fat tissues, with rows showing circRNAs and columns showing tissues. (**D**,**E**) KEGG pathway analysis of DE circRNAs in the skeletal muscle vs. subcutaneous fat tissues. The top 10 enriched KEGG pathways ranked by *p*-values are shown.

Studies have shown that adipose tissue may be involved in the regulation of fat deposition in skeletal muscle indirectly [27,28]. To identify the key circRNAs regulating differences in lipid anabolism, we estimated the expression level of, and performed differential expression analysis on, circRNAs in muscle and adipose tissue. We calculated the RPM value of all circRNAs identified in muscle and adipose tissue and found that 17,244 and 15,929 circRNAs were uniquely expressed in muscle and adipose tissue, respectively, and 12,719 circRNAs were co-expressed in both tissues (Figure 4A). We further identified the DECs between muscle and fat tissue. Among these DECs, compared with adipose tissue, 562 were upregulated and 609 were downregulated in the muscle library (Figure 4B and Supplementary Table S10). In order to further explore the expression pattern of DECs between muscle and adipose tissue, we performed a hierarchical cluster analysis and generated an expression heat map of DECs. The results showed that these circRNAs have obvious differential expression patterns between muscle and adipose tissue (Figure 4C). KEGG pathway enrichment analysis of these upregulated DE circRNAs revealed that host genes were mainly involved in glycogen metabolism-related signaling pathways (e.g., insulin signaling pathway, starch and sucrose metabolism and glucagon signaling pathway), muscular function maintenance and development-related signaling pathways (e.g., hypertrophic cardiomyopathy (HCM), TGF-beta signaling pathway, and AMPK signaling pathway) (Figure 4D). However, the host genes of downregulated DE circRNAs are mainly involved in inflammatory diseases (e.g., ErbB signaling pathway and Fcγ-R-mediated phagocytosis signaling pathway) and fatty acid biosynthesis (Figure 4E). Therefore, these DE circRNAs are potential candidates for the regulation of skeletal muscle development and fat deposition.

### 3.5. Construction of the circRNA-miRNA-mRNA Co-Expression Networks through Time-Series Analysis

**Figure 5:**
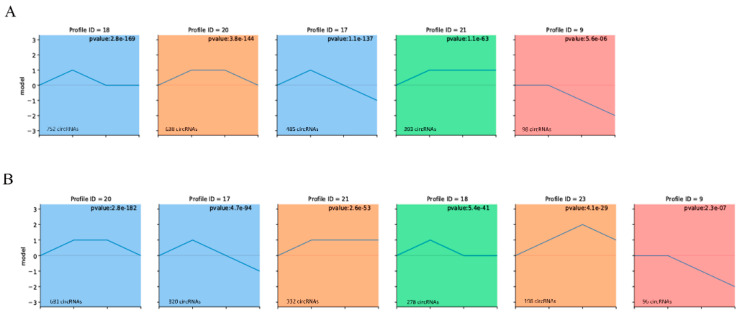
Time-series modules of circRNAs and construction of ceRNA network. Time-series modules of circRNAs in skeletal muscle (**A**) and subcutaneous fat (**B**). Numbers in the top indicate module number. Numbers in top right corner indicate the statistically significant *p* value. Numbers in lower left corners indicate numbers of circRNAs in each module. The colored profiles are indicated by different colors (Bonferroni-adjusted *p* values ≤ 0.05). The *p* value is sorted from small to large. If the profile is the same color, indicating that these profiles belong to the same cluster.

**Figure 6:**
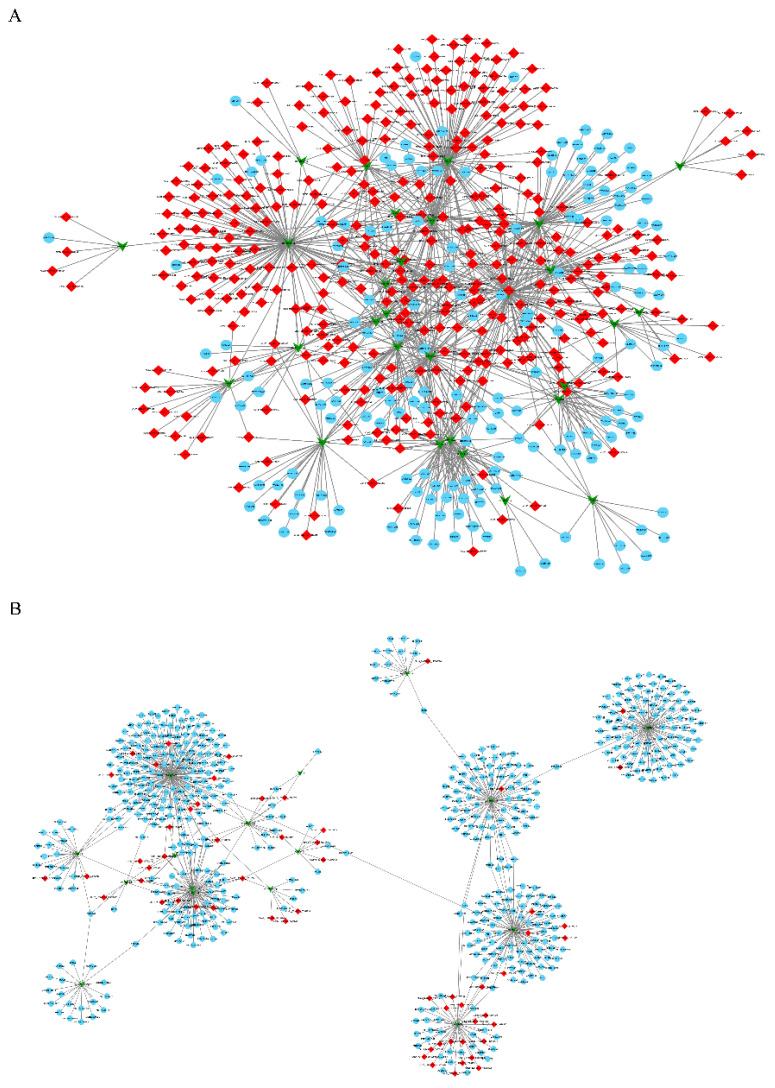
A view of interaction among circRNAs–miRNAs–mRNAs in skeletal muscle (**A**) and subcutaneous fat (**B**). Red diamond nodes represent circRNAs; blue circle nodes represent mRNAs; and green arrow nodes represent miRNAs.

According to the dynamic expression patterns of the circRNAs across the four developmental stages, we found that all the identified circRNAs were classified into four cluster profiles in the muscle and fat. Each tissue included at least five significantly enriched model profiles (Figure 5A,B). Finally, we observed colored modules and only considered the largest module. Functional enrichment analysis showed that the largest module in the two tissues was enriched in a variety of biological processes. Some of these processes were related to signal transduction, cell growth and death, amino acid metabolism, and lipid metabolism (Supplemental Table S11). Previous studies have shown that circRNAs act as miRNA sponges and indirectly regulate gene transcription. To explore whether there are circRNAs with functional correlations or regulatory relationships between circRNAs that are strongly correlated with time points, we performed STEM analysis of miRNAs and mRNAs (Figure S2). In the circRNA STEM analysis, we selected the circRNAs in the colored modules to predict the miRNA and obtained the intersection with the miRNAs that are significant in the miRNA STEM analysis. Next, target analysis was performed with mRNAs that are significant in mRNA STEM analysis. We screened 2384 (fat) and 2672 (muscle) circRNA-miRNA-mRNA network pairs related to developmental time changes (Supplemental Table S12). More importantly, we identified numerous circRNA network pairs related to cell cycle, cell differentiation, fatty acid biosynthesis metabolism markers, such as *CDK16*, *MYH3*, *ACSL3*, *ACSL5*, *APOA4*, and *FADS* (Figure 6A,B). This result indicates that these ceRNA networks may play an important role in the regulation of adipogenesis and metabolism during pig growth periods.

### 3.6. Verification of Identified circRNAs

**Figure 7:**
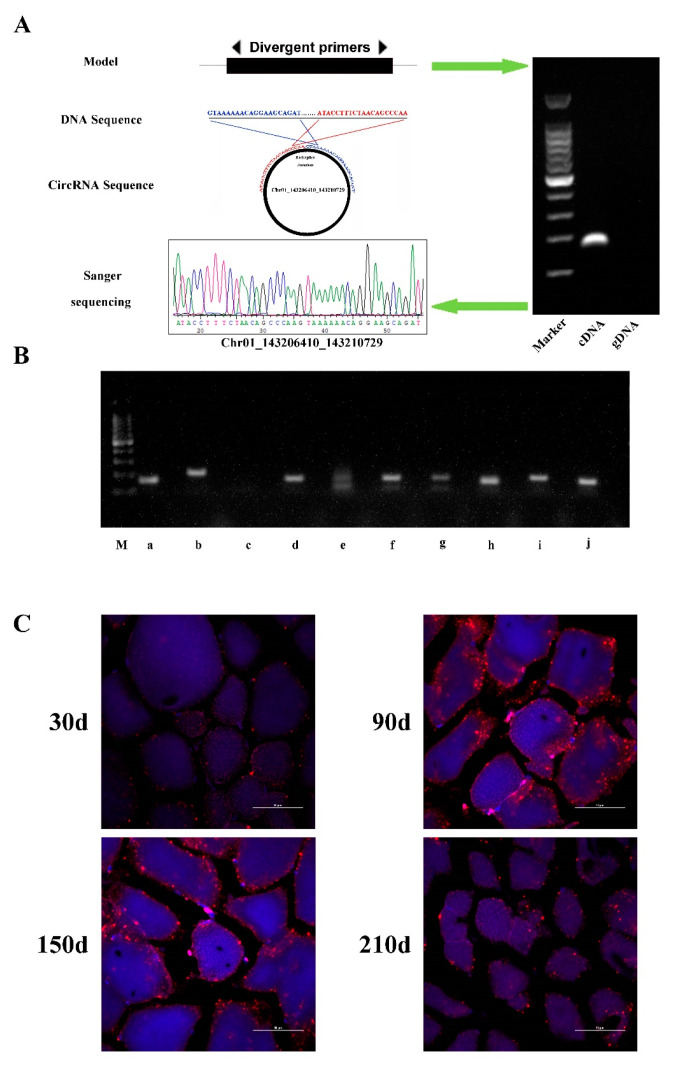
CircRNAs validation by PCR and Sanger sequencing. (**A**) A representative example circRNA-1687 and (**B**) nine other circRNAs were showed in the figure. Successfully validated circRNAs:a, Scaffold155_4811844_4828350; b, Scaffold180_2562774_2570664; d, Chr12_ 55773288_ 55775037; e, Chr02_159842602_ 159843286; f, Chr01_176448874_ 176466186; g, Chr01_ 143206410_143210729; h, Chr04_ 99000917_ 99006790; i, Chr12_ 27312206_ 27323763; J, Chr11_12201402_12205677; not successfully validated circRNA: c, Chr18_ 45729590_ 45744165; (M, 100bp DNA ladder). Detailed information can be found in Table S13. Black arrows represent the PCR amplification orientation of the divergent primer. (**C**) Subcellular localization of Chr01_143206410_143210729in the skeletal muscle at four development time points. Subcellular localization was visualized using panomics probes for high-resolution ISH and DAPI for nuclear localization. Scale bars:50 μm.

In order to verify the accuracy of the circRNAs identified in the circRNA-Seq analysis, we performed reverse transcription RT-PCR and Sanger sequencing experiments on randomly selected 10 circRNAs. A pair of divergent primers are designed for each circRNA, and both cDNA and gDNA (negative control) were used as templates for PCR amplification (Figure 7 and Supplemental Table S13). Among the 10 circRNAs, 9 were successfully confirmed (90%), suggesting the reliability of our circRNA identification results. In addition, as an alternative visualization method, we employed fluorescence in situ hybridization (FISH) for higher resolution subcellular localization of Chr01_143206410_143210729. At four time points of development, the Chr01_143206410_143210729 signal may be related to both the nucleus and cytoplasm. Although signal strength cannot be utilized as a quantitative measure of circRNA abundance, we detected greater signal strength at 90 d and 150 d, which is consistent with the Chr01_143206410_143210729 sequencing data in muscle (Figure 4E and Figure 7C).

## 4. Discussion

### 4.1. Ningxiang Pig Circular RNAs

CircRNAs have been reported to be abundant in animal transcriptomes, and a large number of circRNAs have been identified in humans [29], mice [21], and nematodes [30]. Pigs are important farm animals that provide meat for humans and represent an important animal model for medical research, and increasing numbers of studies have been conducted on pigs in recent years. Liang et al. [19] identified 149 circRNAs that may be related to muscle growth in the analysis of nine organs and three developmental stages of the Guizhou mini pig and found that the host genes of these circRNAs were significantly involved in muscle development and function. These researchers also constructed the first public S. scrofacirc RNA database [19]. Morten et al. [31] depicted the expression of circRNA in five porcine brain tissues at six developmental time points of the fetuses, constituting the first report of circRNA in the development of large animal brains. An unbiased analysis identified a highly complex regulation pattern of thousands of circRNAs with a distinct spatiotemporal expression profile, suggesting the important function of circRNAs in mammalian brain development. Accurate timing of gene expression is highly important for the developmental stage. Because circRNAs are expressed in a highly spatiotemporally specific manner, it is important to conduct research on circRNAs in mammals in different tissues, conditions, and developmental stages. Ningxiang pig is known for their delicious meat, but the regulatory mechanisms that control this characteristic have not been well studied. To study the spatiotemporal expression patterns of circRNA on lipid synthesis and metabolism in Ningxiang pig tissues, we performed total RNA sequencing across skeletal muscle and subcutaneous fat at four developmental stages (30 d, 90 d, 150 d and 240 d after birth). Compared with the reference genomes of other pig species, the results from our work on Ningxiang pig genome assembly in our institution were robust (data not published). The mapping rate of each sample exceeded 92%, and the percentage of Q30 bases was more than 95% (Table 1). We identified 45,892 circRNAs in all evaluated tissues. The identified circRNAs had similar distribution trends in chromosome, number, and length among the three tissues. As indicated in a previous study [19], more circRNAs were generated from chromosome 1, chromosome 6 and chromosome 13 than from the other chromosomes, and the largest number was derived from exons. In addition, the number of circRNAs identified at 90 d was the largest, and the number of uniquely expressed circRNAs at 90 d was considerably greater than that at other time points. We also identified tissue-specific circular RNAs, which provided important information regarding their functions. For example, we identified 15,929 and 17,244 tissue-specific circRNAs in muscle and fat, respectively (Figure 1C). These findings suggest that specifically expressed circRNAs may play specific roles at specific times during the development of tissues or organs. These findings suggest that specifically expressed circRNAs may play specific roles at specific times during the development of tissues or organs. This possibility is consistent with the findings of other studies, indicating that circRNAs have tissue-/stage-specific expression differences [31,32,33]. Subsequently, the identification of circRNAs by grouping statistics indicated that 1786 and 3116 circRNAs with significant differential expression in Ningxiang pig muscle and adipose tissue, respectively.

### 4.2. Circular RNAs in Muscle and Fat

Because the number of muscle fibers has been determined before birth, skeletal muscle mass increases through hypertrophy during postnatal development, and a similar process may be induced in adult skeletal muscle in response to contractile activity [34]. Each skeletal muscle in an animal contains a different type of fiber. Type I muscle fibers are rich in mitochondria and have a higher oxidative metabolism, while type IIB fibers have fewer mitochondria, and the glycolytic metabolism capacity is higher [35]. However, the role of circRNAs in skeletal muscle development is unknown. This study identified circRNAs that were abundantly expressed in muscle, including Chr05_81425549_81426478, Chr15_85761324_85761662, Scaffold180_2562774_2570664, Chr02_159842602_159843286, and Chr12_ 55773288_ 55775037 (Figure 2E). The contractile protein troponin I (TnI) is a component of the troponin complex located on the striated muscle filaments, and is involved in calcium-mediated contraction and relaxation. Vertebrate TnI has evolved into three isotypes encoded by three homologous genes, which are expressed under muscle type-specific and developmental regulatory mechanisms [36,37]. Among these genes, TNNI2 (fast-twitch skeletal muscle isoform, named TNI-fast) is a specific protein of the fast muscle fiber type. During muscle development, Chr02_159842602_159843286, produced by the host gene *TNNI2*, was abundantly expressed at 30 d. It has been reported that the change in *MYBPC1* abundance is related to the production of slow-twitch muscle fibers [38,39]. Interestingly, we observed a peak expression of Chr05_81425549_81426478 transcribed by myosin binding protein C1 (*MYBPC1*) as a host gene in the developing 90 d pig muscle. Chr15_ 85761324_ 85761662, produced by the host gene titin (*TTN*), was demonstrated to be expressed at 30 d and reached its peak at 210 d. It has been reported that *TTN* is related to intramuscular fat deposition [40], and recent studies have found that the circular RNA *TTN* acts as a miR-432 sponge to facilitate the proliferation and differentiation of cattle myoblasts via the IGF2/PI3K/AKT signaling pathway [41]. Interestingly, we found 755 circRNAs transcribed from the *TTN* gene. Among them, 643 circRNAs were significantly associated with 17 circRNAs from human *TTN* genes, such as has_circ_0057213, has_circ_0057224, has_circ_0057213, has_circ_0057220 and has_circ_0057221, etc. However, only 9 porcine circRNAs and mmu_circ_0010191 from mouse *TTN* gene were highly conserved. *TTN* is a gene known to have highly complex alternative splicing. It has always been considered as a mechanical protein in muscle cells, and its main function is to act as a molecular spring in the muscle [42]. We found that some of the circRNAs transcribed from the *TTN* gene are not only highly expressed in abundance but also dynamically regulated during muscle development, and some are uniquely expressed at certain time points (Supplemental Table S2). In addition, the most abundant circRNAs expressed in muscles are also derived from members of the *MYH* myosin superfamily, such as *MYH2* and *MYH6*. Recent studies have shown that these genes are associated with skeletal muscle development and influence the quality of pork [43]. These findings suggest that many circRNAs exhibit different expression patterns during muscle development, and changes in the abundance of these circRNAs may affect muscle fiber composition, thereby affecting the properties of meat. Notably, pathway analysis of the genes giving rise to circRNAs peaking at 30 d indicated a significant predominance of genes associated with fatty acid biosynthesis, primary bile acid biosynthesis, unsaturated fatty acid biosynthesis, and peroxisome biosynthesis. The impact of circRNAs on the biosynthesis of unsaturated fatty acids will be of great concern and may be related to the higher proportion of unsaturated fatty acids in the diet of piglets. Malic enzyme 1 (*ME1*) plays an important role in the Krebs cycle for energy metabolism. The mRNA of *ME1* was observed to be more abundant in obese Rongchang pigs than in lean Landrace pigs. Furthermore, mRNA abundance changes in ME1 have a marked significant positive correlation with adipocyte volume across the six adipose tissue types [44]. Chr01_176448874_176466186 is produced by the host gene *ME1*, which is abundantly expressed in adipose tissue (Figure 3E). The expression of Chr01_ 176448874_ 176466186 was shown to increase from 90 d, peaking at 150 d and subsequently, with fluctuations, decreasing until 210 d. This gene is surmised to be closely related to adipocyte development and deposition. In addition, we found that Chr01_143206410_143210729 was abundantly expressed in muscle and fat but presented different expression patterns. For example, the expression of Chr01_ 143206410_ 143210729 in developmental adipose tissue was shown to increase from 30 d, peak at 150 d and subsequently, with fluctuations, decrease towards 210 d. However, the expression of Chr01_143206410_143210729 in muscle peaked at 90 d and decreased gradually thereafter. Although the host gene SAFB-like transcription regulator (SLTM) of Chr01_143206410_143210729 is rarely reported, its homologous family member SAFB1 can interact with a variety of other nuclear receptors and inhibit their transcriptional activity, including PPARγ, FXRα, RORα1, PPARα, PPARβ, VDR, SF1, and LRH-1 [45]. SAFB1 has also been shown to regulate the expression of SREBP-1c. SREBP-1c is a bHLH transcription factor that controls lipogenesis in the liver. It is induced by excessive nutrient supply to promote the conversion of glucose into fatty acids and triglycerides to store excess energy [46]. SLTM has 34% overall identity with SAFB1. SLTM contains SAP and RRM domains, and their similarities with SAFB1 are 60% and 70%, respectively [47,48]. The existence of these shared domains indicates that SAFB1 and STLM have at least some common functions; however, it is reasonable to suspect that the circularization of these genes may play important roles in additive, synergistic, or potentially antagonistic functions exhibited by different family members, but these speculated interactions require follow-up experimental verification.

### 4.3. Network of circRNA, miRNA and mRNA

Time-series analysis showed that certain differential circRNAs are dynamically expressed during skeletal muscle and adipose tissue development. Although the molecular function of circRNAs has not been fully elucidated, the current research shows that in addition to the function of circRNAs in regulating host gene transcription, protein binding and translation, further research is needed to determine the role of miRNA sponges [49,50]. Competing endogenous RNAs (ceRNAs) are widely present in muscle and fat. For example, circular RNA *SNX29* sponges miR-744 to regulate the proliferation and differentiation of myoblasts by activating the Wnt5a/Ca signaling pathway [51]. CircSAMD4A regulates preadipocyte differentiation by acting as a miR-138-5p sponge and thus increasing *EZH2* expression [52]. CircRNA_0046366 inhibits hepatocellular steatosis by abolishing the miR-34a-dependent inhibition of *PPARα* [53]. In our study, STEM analysis and ceRNA analysis were combined to screen out the interaction network pairs closely related to time point changes. For example, G protein signaling 9 (*RGS9*) is the targeted gene of ssc-miRNA-7137-3p, while the miRNA targeted eight circRNAs (Chr15_57751128_ 57770746, Chr14_24730872_24760271, Chr03_19654503_ 19689919, Chr11_ 10784978_ 10824218, Chr06_124537421_ 124559486, Chr08_82174982_82248693, Chr07_23444137_23455147, Chr12_24249907_ 24258995) (Figure 6A) in the muscle network diagram. As reported, *RGS 9* knockout mice showed higher body weight and greater fat accumulation than wild-type mice [54,55]. In addition, we also identified numerous well-known key markers in the muscle ceRNA networks, e.g., *CDK16*, *CYC1*, *MYH3*, *HDC*, and *E2F1* for muscle cell growth and differentiation and *FAAH*, *PLIN3*, *PNPLA2*, and *GAS7* for lipid synthesis and metabolism [56,57,58], suggesting that these ceRNAs networks may play critical roles during muscle growth and development processes and providing ideas for further research studying muscle development and intramuscular fat deposition.

The differentiation of adipocytes is a highly complex biological process. While regulated by a series of transcription factor cascades, various cytokines can also participate in initiating and regulating the differentiation of adipocytes and maintaining cell characteristics through complex signal transduction. Adipose tissue is also an active endocrine organ that can secrete some hormones or cytokines (e.g., resistin, tumor necrosis factor-α and complement D) to participate in the immune response and treatment of obesity or cardiovascular diseases and other physiological and pathological processes [59,60]. However, these functions may be inseparable from the regulation of circRNAs. For example, in our study, Chr15_78588493_ 78637426, produced by *TLK1* as the host gene, was abundantly expressed in adipose tissue, starting at 30 d and decreasing after peaking at 150 d (Figure 3E). Tousled-like kinase 1 (*TLK1*) is a serine/threonine protein kinase that is implicated in chromatin remodeling, DNA replication and mitosis [61]. Combined with the joint analysis results of SETM and ceRNA, it was found that among the predicted target miRNAs of circRNA *TLK1*, only miRNA-204 was significantly expressed in the STEM analysis of miRNA. Among the target mRNAs of miRNA-204, only four mRNAs (*LALMA4*, *COL6A1*, *ABCD2*, *ELMOD3*) were significantly expressed in mRNA STEM analysis (Supplemental Table S12). It has been reported that lamininα4 (*LAMA4*) is located in the extracellular basement membrane that surrounds each individual adipocyte and affects the structure and function of adipose tissue in a depot-specific manner. Compared with wild-type mice, *LAMA4*^−/−^ mice showed higher energy expenditure at room temperature and when exposed to a cold challenge. In addition, the mice had decreased adipose tissue mass and altered lipogenesis in a depot-specific manner [62,63]. Interestingly, some scientists believe that obesity is an inflammatory condition that is associated with increased extracellular matrix (ECM) gene expression, whereas the collagen 6A1(*COL6A1*) gene is the primary gene in the ECM [64]. The ATP-binding cassette transporter *ABCD2* (*D2*) is a peroxisomal protein whose mRNA is highly expressed in fat and upregulated during adipogenesis. There are also articles reporting that *D2* can not only promote the oxidation of long-chain monounsaturated fatty acids (LC-MUFAs) but also inhibit the accumulation of dietary erucic acid (EA, 22:1ω9) in fat cells [65,66]. These findings indicate that the same circRNA may play different roles in fat development and function. These results indicate that these ceRNA networks are active throughout tissue development. Although these in silico results should be investigated further in vivo, these results imply functional relatedness or a regulatory relationship between circRNAs and miRNAs during tissue development.

Furthermore, 12,719 identified circRNAs were co-expressed in both muscle and fat. Among them, we found a large number of co-expressed DE circRNAs in fat and muscle. Compared with adipose tissue, host genes of upregulated circRNAs in muscle are related to glucose metabolism and insulin signaling pathway, such as Chr01_127164838_127186030, Chr14_3923800_3930511, Chr01_127171889_127186030, Chr06_21251698_21261868, etc. Their host genes were glycogen synthase 1(*GYS1*), acetyl-CoA carboxylase alpha(*ACACA*), glucosidase alpha, neutral C(*GANC*) and phosphoglucomutase 1(*PGM1*), respectively. It is reported that skeletal muscle is an important organ regulating glucose and lipid metabolism, and it is an insulin-dependent glycogen intake tissue. When fat deposition in skeletal muscle is excessive, glycogen intake in skeletal muscle will be affected. Lipid deposition in skeletal muscle fibers is closely related to the occurrence and development of peripheral insulin resistance and type 2 diabetes mellitus [67]. Therefore, these circRNAs can serve as potential targets for the study of muscle glucose metabolism and insulin resistance. In addition, we also found some differences in circRNAs related to fatty acid synthesis between muscle and fat, such as Chr15_46857316_ 46859616 and Chr14_120982040_120985676, whose host genes were derived from acyl-CoA synthetase long chain family member 1(*ACSL1*) and acyl-CoA synthetase long chain family member 5(*ACSL5*), respectively. Differential expression of these circRNAs may be one of the reasons for the differences in fatty acid synthesis between fat and muscle.

## 5. Conclusions

In summary, we first revealed the expression profile of circRNAs in four pig growth stages. A large number of differentially expressed circRNAs between muscle and fat were identified, and KEGG pathway enrichment analysis of these DE circRNAs revealed that host genes were mainly involved in metabolism-related signaling pathways, and fatty acid anabolism. The highly expressed circRNAs related to muscle development and fat deposition were verified and were consistent with RNA-seq results. The results provide a significant expression database for studying circular RNAs during pig muscle growth and fat deposition.

## Figures and Tables

**Table 1:** Sequencing basic data of four different developmental stages of Ningxiang pig two tissues.

Terms	NX30d		NX90d		NX150d		NX210d	
	Muscle	Fat	Muscle	Fat	Muscle	Fat	Muscle	Fat
Raw reads number	65606883	57278618	50488816	53672934	53929255	45120232	56107588	58206111
Clean reads number	64137048	56517482	48897656	52575561	52912480	44451010	55237321	57266380
Clean reads rate	97.76%	98.67%	96.85%	97.96%	98.11%	98.52%	98.45%	98.39%
Clean Q30 bases rate	95.42%	95.02%	95.35%	95.48%	95.35%	95.48%	95.63%	95.28%
Mapped reads	120386916	106157588	91329628	97298423	99877320	81793231	104372240	105818225
Mapping rate	93.85%	93.90%	93.38%	92.55%	94.38%	92.00%	94.48%	92.41%

The values represent the reads and proportion that were compared to those in the Ningxiang pig reference genome (PRJNA531381).

## Data Availability

The data set generated and analyzed in this study can be obtained in the repository, as follows: Whole transcriptome high-throughput sequencing data access code: PRJNA721288 (https://www.ncbi.nlm.nih.gov/bioproject/PRJNA721288/, accessed on 12 April 2021).

## Supplementary Materials

The following are available online at https://www.mdpi.com/article/10.3390/biology10090841/s1, Figure S1: KEGG pathway analysis. KEGG analysis of DE circRNAs in the 30 d vs. 90 d, 30 d vs. 150 d, 30 d vs. 210 d, 90 d vs. 150 d, and 150 d vs. 210 d groups. The top 10 enriched KEGG pathways ranked by P-values are shown.vs. means versus. Figure S2: Time-series modules of miRNA (A) and mRNA (B) in skeletal muscle and subcutaneous fat. The colored profiles are indicated by different colors (Bonferroni-adjusted *p* values ≤ 0.05). The *p* value is sorted from small to large. If the profile is the same color, indicating that these profiles belong to the same cluster. Table S1: List of identified circRNAs during the development of the two tissues. Table S2: Blast analysis Ningxiang pig circRNAs have orthologs in mice and humans, respectively. Table S3: List of differentially expressed (DE) circRNAs for five comparison groups in muscle. Table S4: List of KEGG pathways enrich of differentially expressed (DE) circRNAs from five comparison groups in three tissues. Table S5: List of common differentially expressed (DE) circRNAs for three developmental stages (90d, 150d, and 210d) vs30d in muscle. Table S6: List of top 20 circRNAs expressed in muscle. Table S7: List of differentially expressed (DE) circRNAs for five comparison groups in fat. Table S8: List of common differentially expressed (DE) circRNAs for three developmental stages (90d, 150d, and 210d) vs30d in fat. Table S9: List of top 20 circRNAs expressed in subcutaneous fat. Table S10: List of differentially expressed (DE) circRNAs between skeletal muscle and subcutaneous fat. Table S11: KEGG pathways enrich of circRNAs list in model profiles of STEM. Table S12: The ceRNA networks of circRNAs and their targeted miRNAs and mRNAs. Table S13: The primers used in this study.

## Abbreviations

ncRNA, noncoding RNA; lncRNA, long noncoding RNA; miRNA, microRNA; circRNAs, circular RNAs; 30 d, 30 day; 90 d, 90 day; 210 d, 210 day; vs., versus; DEGs, Differentially expressed mRNAs; ceRNA, Competitive endogenous RNA; DECs, Differentially expressed circRNAs; FDR, False discovery rate; TPM, Transcripts Per Kilobase of exon model per Million mapped reads; KEGG, Kyoto Encyclopedia of Genes and Genomes; STEM, Short Time-Series Expression Miner; RPM Reads per million mapped reads; RNA-Seq, RNA sequencing; CIRI 2, CircRNA identifier.
