# Supplementary figures and images for: Spatiotemporal Regulation and Functional Analysis of Circular RNAs in Skeletal Muscle and Subcutaneous Fat during Pig Growth
Source: Biology (Basel). 2021 Aug 30;10(9):841. doi: 10.3390/biology10090841 (PMC8465536; doi:10.3390/biology10090841)

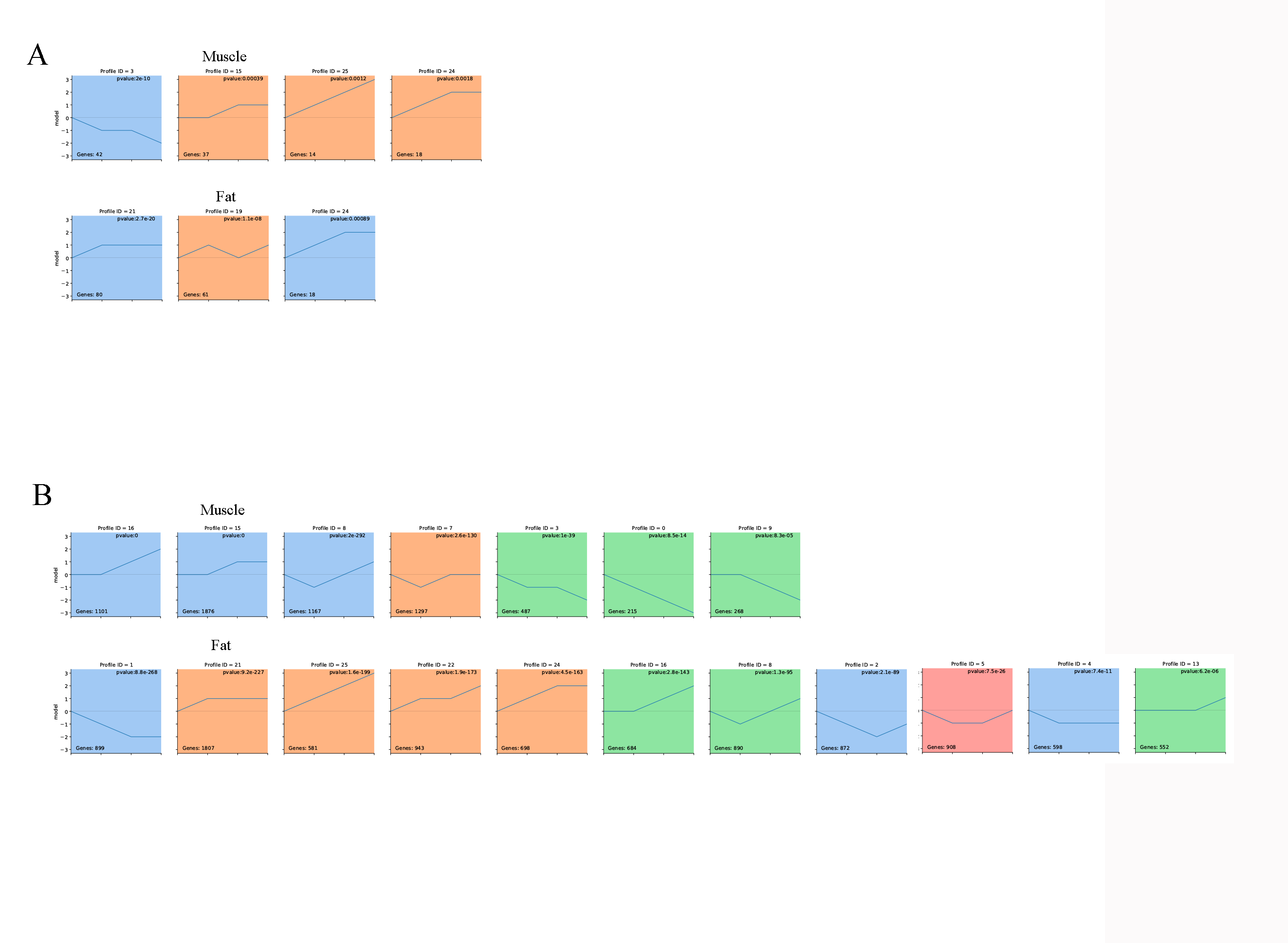

Supplement: Supplementary file 1 [file biology-10-00841-s001.zip › Supplemental Figure S2.tif]
